# The role of ADAM10 in astrocytes: Implications for Alzheimer’s disease

**DOI:** 10.3389/fnagi.2022.1056507

**Published:** 2022-11-30

**Authors:** Richard J. Elsworthy, Eric J. Hill, Connor Dunleavy, Sarah Aldred

**Affiliations:** ^1^School of Sport, Exercise and Rehabilitation Sciences, University of Birmingham, Birmingham, United Kingdom; ^2^Centre for Human Brain Health, University of Birmingham, Birmingham, United Kingdom; ^3^School of Biosciences, Aston University, Birmingham, United Kingdom

**Keywords:** astrocyte, ADAM10 (a disintegrin and metalloprotease 10), inflammation, Alzheimer’s disease, amyloid-beta

## Abstract

Much of the early research into AD relies on a neuron-centric view of the brain, however, evidence of multiple altered cellular interactions between glial cells and the vasculature early in AD has been demonstrated. As such, alterations in astrocyte function are widely recognized a contributing factor in the pathogenesis of AD. The processes by which astrocytes may be involved in AD make them an interesting target for therapeutic intervention, but in order for this to be most effective, there is a need for the specific mechanisms involving astrocyte dysfunction to be investigated. “α disintegrin and metalloproteinase” 10 (ADAM10) is capable of proteolytic cleavage of the amyloid precursor protein which prevents amyloid-β generation. As such ADAM10 has been identified as an interesting enzyme in AD pathology. ADAM10 is also known to play a role in a significant number of cellular processes, most notable in notch signaling and in inflammatory processes. There is a growing research base for the involvement of ADAM10 in regulating astrocytic function, primarily from an immune perspective. This review aims to bring together available evidence for ADAM10 activity in astrocytes, and how this relates to AD pathology.

## Introduction

Alzheimer’s disease (AD) is the most common type of dementia accounting for 50–75% of all dementia cases worldwide (Prince et al., 2015). Despite an age-specific lowering of AD risk in some high-income countries, the worldwide prevalence of AD is predicted to rise rapidly, doubling every two decades (Langa, 2015). The progression of AD can have devasting effects on a person’s quality of life and is ultimately fatal. Therefore, it is critical that disease-modifying treatments are developed targeting early pathogenic processes, with the aim of delaying the onset or progression of AD symptoms. The hallmark pathological features of the AD brain are described as the accumulation of Amyloid-β (Aβ) plaques and intracellular hyperphosphorylated Tau tangles, in addition to synapse loss and neuronal degeneration (Hampel et al., 2021). The accumulation of Aβ species is hypothesized to be a driving force in the development of AD (Selkoe and Hardy, 2016). However, there is evidence to the contrary, such as the presence of Aβ in cognitively healthy individuals, and the lack of correlation between amyloid plaque load and cognitive function (Morris et al., 2014; Gómez-Isla and Frosch, 2022). What is widely accepted, is that the development of AD begins much earlier than a clinical diagnosis is reached, preceding the onset of symptoms by 20–30 years (Selkoe and Hardy, 2016; Pfeil et al., 2021). This prodromal stage of AD is perhaps the most opportune timepoint for effective interventions, thus identifying disease processes in this period is an area of intense research. Much of the early research into AD relies on a neuron-centric view of the brain, however, evidence of multiple altered cellular interactions between glial cells and the vasculature early in AD has been demonstrated (De Strooper and Karran, 2016).

**Figure 1:**
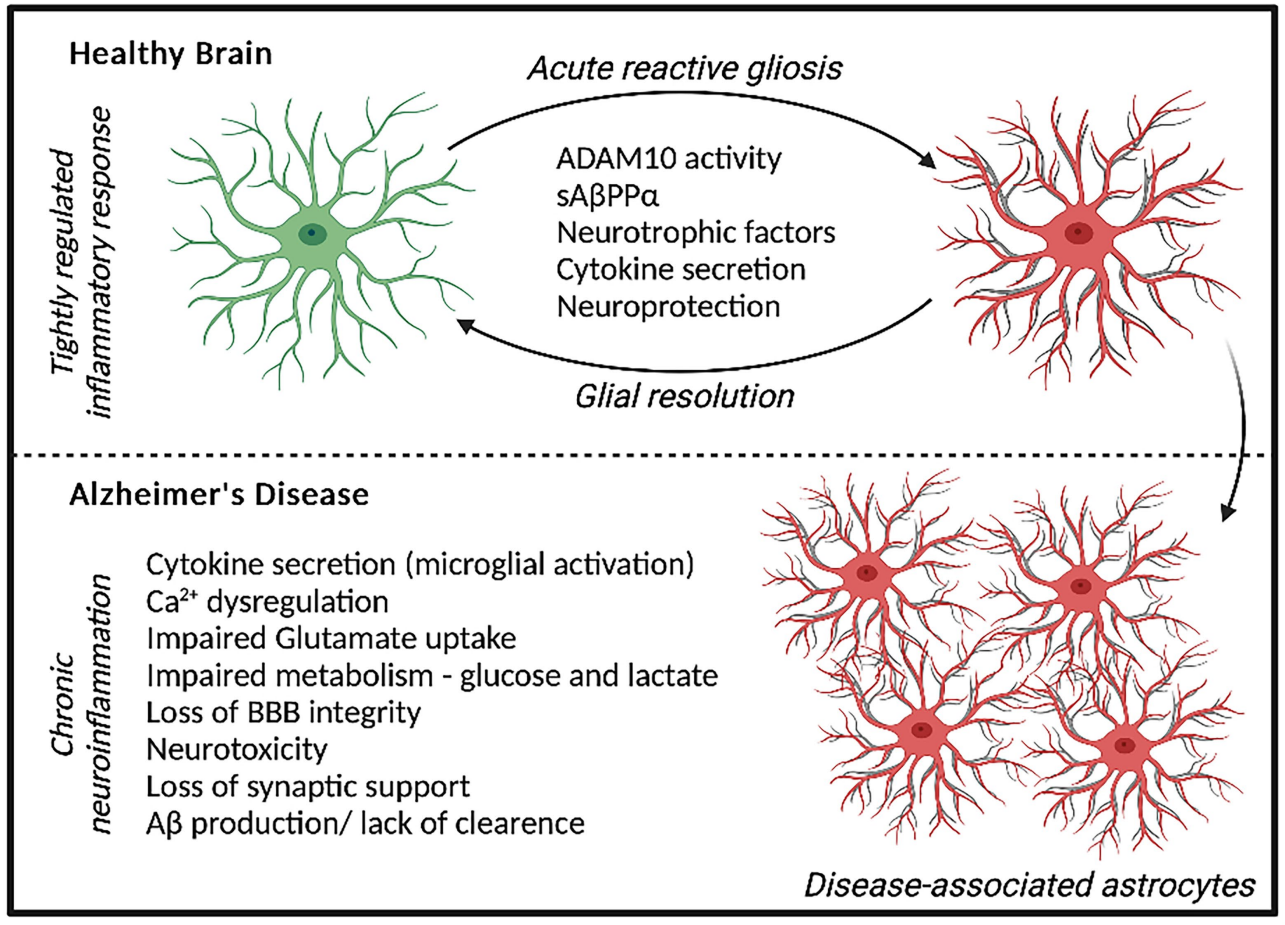
Astrocyte response to acute stressors elicits a neuroprotective phenotype and localized inflammatory state that can be resolved over time. In AD, a chronic neuroinflammatory state is present whereby prolonged astrocyte dysfunction is evident, contributing to neurodegeneration and AD pathology.

Astrocyte dysfunction is becoming widely recognized to contribute to the pathogenesis of AD, showing both adaptive and adverse profiles effecting neurodegeneration (Monterey et al., 2021). The diverse functions of Astrocytes include regulating synaptogenesis, cellular signaling, neurotransmitter buffering and ion homeostasis in addition to providing key metabolic support to neuronal cells (Khakh and Deneen, 2019). Astrocytes are also important for protecting against and repairing neuronal damage can undergo reactive gliosis when responding to inflammatory stimulus (Li et al., 2019). Reactive astrocytes secrete growth factors, cytokines and gliotransmitters to promote the resolution of acute injury, which diminished once resolved. However, in AD, chronic inflammatory conditions can persist increasing the risk of neuronal dystrophy (Figure 1; Monterey et al., 2021). Recent data has demonstrated that disease-specific astrocytes are linked to a proinflammatory profile. These inflammatory-linked states, also termed as astrocyte reactivity, form a heterogenous and functionally diverse response effecting cell morphology, molecular expression, and cellular function in response to a changing environment (Sofroniew, 2020). Cytoskeletal remodeling, often characterized by changes in Glial fibrillary acidic protein (GFAP) levels, neuroinflammation, redox balance, lipid and protein metabolism have all been identified to be altered in astrocytes from the AD brain (Viejo et al., 2022). Although previously considered as simply supporting cells, astrocytes are recognized as having increasingly important physiological roles in the brain and represent a highly heterogenous cell population (Garwood et al., 2017). In 5xFAD mice, distinct astrocyte populations could be subtyped into six clusters based on GFAP expression, with clusters 1 and 2 reflecting homeostatic astrocytes and cluster 6 representing a disease state (Habib et al., 2020). ‘High GFAP astrocytes’ states were associated early in the AD pathology prior to cognitive impairment and increased with disease duration. Interestingly, wild-type mice showed a trajectory toward high GFAP cluster astrocytes with aging (Habib et al., 2020). In support of this, increased GFAP reactivity was shown to be present in both aged mice and in the earlier development of double transgenic (B6.152H) mouse line, suggesting FAD mutations may represent an accelerated aging phenotype (Leparulo et al., 2022). In humans elevated GFAP has been identified in both cognitively healthy aging and AD, reflecting cognitive ability irrespective of diagnosis (Bettcher et al., 2021). Interestingly however, GFAP is elevated in cognitively healthy individuals with higher Aβ loads, suggesting there may be some link between astrocytic damage and Aβ accumulation in pre-symptomatic AD (Chatterjee et al., 2021). This astrocytic response may primarily represent a defensive state, whereby relatively mild isomorphic gliosis occurs. Astrocytes are also critical for providing neuronal support, actively modulating processes at the synapse and maintaining the blood brain barrier (Allen and Eroglu, 2017). Perisynaptic astrocytes can rapidly remove neurotransmitters such as glutamate from the synaptic cleft, preventing accumulation and thus, neurotoxicity. This can be recycled back to neurons in the form of glutamine to support synaptic transmission. Further metabolic support can be provided by astrocytes to neurons, by astrocytic lactate shuttling (Pellerin et al., 1998).

The processes by which astrocytes may be involved in AD pathogenesis make them an interesting target for therapeutic intervention, but in order for this to be most effective, there is a need for the specific mechanisms involving astrocyte dysfunction to be investigated. There has been a mounting evidence base for the involvement of “α disintegrin and metalloproteinase” 10 (ADAM10) in AD progression, therefore this review aims to bring together available evidence for ADAM10 activity in astrocytes, and how this relates to AD pathology.

### ADAM10 in Alzheimer’s disease

The metalloproteinase, ADAM10 belongs to a family of membrane-anchored proteases capable of ‘ectodomain shedding’, a process of cleaving the extracellular domain of close-proximity targets (Hsia et al., 2019). ADAM10 is known to cleave a number of proteins from cell adhesion molecules to membrane receptors and is ubiquitously expressed in both peripheral and central tissues (Hsia et al., 2019). The ADAM10 gene is localized on chromosome 15 and mutations effecting the enzymatic activity of ADAM10 are associated with an increased risk of AD (Kim et al., 2009; Jansen et al., 2019). Mutations affecting the pro-domain of ADAM10 appear to be responsible for this loss of activity perhaps unsurprisingly, as pro-domain removal is a key step in ADAM10 maturation (Anders et al., 2001). Blocking this process can significantly reduce membrane expression of ADAM10 (Seifert et al., 2021). The trafficking of ADAM10 is also a key process in determining the colocalisation and targeting of ADAM10 to receptors and substrates. This trafficking is thought to be highly regulated by members of the Tetraspanin (TSPAN) family of transmembrane proteins, in particular the TSPAN C8 subgroup (Matthews et al., 2017). Cell type dependent repertoires of TSPAN C8s may elucidate interesting targets for ADAM10 activity regulation, although this is highly speculative at this stage (Seipold and Saftig, 2016).

The importance of ADAM10 function in neurodevelopment is highlighted by the embryonic lethality of ADAM10 deficiency in mice, which is known to interfere with notch receptor signaling. Attempts to understand the maturation, trafficking and regulation of ADAM10 have revealed several key processes that may govern the enzymes interaction with target molecules and are beginning to resolve therapeutic options (Musardo et al., 2022). Similarly, modulation of both serotonin receptor (5HTr) four and six stimulate ADAM10 activity and subsequent sAPPα secretion, which is linked to APP processing and are a target for AD treatment.

As the major α-secretase responsible for APP processing, ADAM10 has been of significant interest in AD (Postina et al., 2004; Kuhn et al., 2010). While the amyloidogenic cleavage of APP by the β-amyloid converting enzyme (BACE-1), followed by y-secretase cleavage, liberates sAPPβ and the Aβ peptide, initial ADAM10 cleavage of APP can prevent BACE-1 interaction and therefore preclude the formation of Aβ (Zhang et al., 2011). Not only this, ADAM10 cleavage of APP liberates sAPPα which is thought to be neuroprotective. Attempts to characterize ADAM10 as a potential peripheral biomarker of AD have revealed several interesting findings, with lower platelet ADAM10 expression in people with AD (Colciaghi et al., 2002, 2004). Platelet ADAM10 activity also correlates with measures of cognitive function, suggesting lower ADAM10 is related to worse cognitive function in people with AD (Manzine et al., 2013, 2014). However, when measuring ADAM10 protein levels in plasma and cerebral spinal fluid, an apparent opposite relationship has been shown, where elevated ADAM10 has been reported in people with AD (Pereira Vatanabe et al., 2021). As ADAM10 is active as a membrane protease it is likely that soluble ADAM10 has undergone ectodomain shedding by other members of the ADAM family, and thus represents a soluble inactive protein (Parkin and Harris, 2009; Pereira Vatanabe et al., 2021). Therefore, elevated soluble levels of ADAM10 may be linked to reduced overall enzyme activity. Although the reasons for this increased ectodomain shedding has not been investigated, it is possible that reduced ADAM10 activity leads to an increase in removal of the enzyme from the cell membrane *via* internalization and secretion in extracellular vesicles thus, increasing extracellular levels (Seifert et al., 2021). As ADAM10 acts primarily at the cell membrane to cleave close-proximity targets (Hitschler and Lang, 2022), the plasma and CSF activity levels may reflect a decreased amount of functionally active ADAM10, therefore, explaining reduced ADAM10 activity in AD. This has led to the idea of increasing, or at least restoring ADAM10 activity, as a therapeutic option for Alzheimer’s disease. Yet, this is not without concern as the involvement of ADAM10-mediated cleavage in propagating inflammatory signaling processes and adhesion molecules could negatively impact other tissue or cell types (Pruessmeyer and Ludwig, 2009). Evidence of these unwanted side effects can be drawn from failed metalloproteinase inhibitors for cancer treatment which had limited specificity to particular substrate processing axis (Wetzel et al., 2017). This has placed an emphasis on the need to understand how ADAM10 activity is related to its localisation and functionality, as controlled modulation will be critical, likely being administered within a particular therapeutic window early in the disease process (Wetzel et al., 2017). Interestingly, limiting ADAM10 endocytosis, which promotes the maintenance of cell surface activity without interfering with canonical activity, may be one avenue to therapeutic intervention (Musardo et al., 2022). Similarly, ADAM10 activity may also play role in our understanding of how non-pharmacological interventions such as exercise, can improve cognitive function. However, before this can be answered, it is critical that we gain a better understanding how ADAM10 activity is regulated in the brain and between different cell types. With a growing acknowledgment of the importance of astrocytes in AD progression, understanding ADAM10 expression and activity may be of great significance.

### ADAM10 activity in astrocytes

Astrocytes are a heterogenous, non-neuronal cell type that perform a plethora of functions in the brain. Several key functions of astrocytes are to assist in the formation, maintenance and remodeling of synapses (Chung et al., 2015). Astrocytes form a “synaptic cradle” around the synapse allowing regulation of neurotransmitters, ion gradients, releasing neuromodulators and providing metabolic support to neighboring cells (Matias et al., 2019). Further regulatory roles of astrocytes in controlling blood–brain barrier permeability and immune signaling have been identified. Astrocytes are key in maintaining homeostatic brain function and play a significant role in neuroprotection in acute brain injury (Bylicky et al., 2018; Mederos et al., 2018). Considering the multitude of functions of astrocytes in maintaining homeostasis within the brain, it is clear that they possess a more significant role in the central nervous system than the ‘glue’ for neuronal structures, and it is perhaps not surprising that their dysfunction is related to a wide-range of neuro-pathologies (Siracusa et al., 2019).

### ADAM10 and inflammatory processes in astrocytes

The contribution of glial cells to inflammatory processes, the expression and secretion of APP processing machinery, and their importance for maintaining the synapse, highlight the potential importance of astrocytes in AD pathology. The role of ADAM10 in many of these processes suggest the enzyme is likely to play an interesting role in astrocyte function (Goetzl et al., 2016; Sofroniew, 2020). Despite the interest in ADAM10 activity and proposed roles of astrocytes in AD pathology, there is relatively little research specifically investigating how ADAM10 is regulated in astrocytes. Much of this research is focused on ADAM10 and cytokine signaling, where stimulation of astrocytes with proinflammatory mediators have been shown to induce ADAM10 ectodomain shedding. In human astrocytes, a combined Interleukin-1β (IL-1β), Tumor Necrosis Factor-α (TNFα) and Interferon-γ (IFNγ) treatment increased ADAM10 mediated Fractalkine (CX3CR1) shedding, which is reported to be neuroprotective (O’Sullivan et al., 2016). Further, exposure of the C6 rat astroglia cell line to acrolein, a product of lipid peroxidation, resulted in increased ADAM10 levels and n-cadherin cleavage. Acrolein treated astrocytes also displayed reduced glutamate uptake, which is a critical function of astrocytes at the synapse to support neuronal function (Park et al., 2020). In mouse models of traumatic brain injury, which are known to induce a potent inflammatory response, ADAM10 expression has been shown to colocalise with GFAP positive cells (Del Turco et al., 2014). The increased expression of ADAM10 as part of reactive plasticity to brain injury may facilitate synaptogenesis (Warren et al., 2011). The activity of ADAM10 is also increased, shown by elevated N-cadherin cleavage which was linked to reactive gliosis (Warren et al., 2012). The relationship between ADAM10 and inflammatory processes appears to be bi-directional. ADAM10 prodomain mutations which have been proposed to increase the risk of AD, are associated with increased gliosis, which would indicate a more reactive astrocytic phenotype (Suh et al., 2013). In support, overexpression of ADAM10 is associated with reduced expression of protein GFAP positive astrocytes, which is indicative of reduced gliosis (Zhu et al., 2021). Interestingly, conditional knock out of ADAM10 in mouse derived neurospheres, which represent a neurodevelopmental timeframe, elevated the ratio of neuronal cells at the expense of astrocytes. In support of this, APP and its secreted sAPPα fragment induce glial differentiation of neural stem cells (Kwak et al., 2010, 2014). This suggests ADAM10 may play a critical role in the proper development of neural progenitor pools and its removal may lead to early neuronal dominant cortical differentiation as astrocyte typical appear much later in development (Jorissen et al., 2010). In later stages of development, it is possible that ADAM10 activity is constitutively lower in astrocytes compared to neurons (Guo et al., 2016), and is instead activated in response to inflammatory stimulus.

### ADAM10 and APP processing in astrocytes

The role of ADAM10 in non-amyloidogenic APP processing has been known for a number of years, although how ADAM10 is regulated in astrocytes is not well characterized. Early evidence characterizing the balance between APP processing pathways in primary astrocytes showed that while non-amyloidogenic processing is evident, it is significantly lower than that of amyloidogenic processing (LeBlanc et al., 1997). However, when compared to primary neurons the total amyloid generation from astrocytes was much lower which was matched by total APP protein levels (LeBlanc et al., 1996). In addition, the APP751 isoform containing Kunitz serine protease inhibitor domain in predominant in astrocytes which differs to the APP695 isoform in neuronal cells (LeBlanc et al., 1997). More recent research into ADAM10 in astrocytes is often limited to investigation of the effect of induced inflammation, which can directly affect APP processing. IL-1α treatment of the U373 astrocytoma has been shown to stimulate non-amyloidogenic APP processing *via* ADAM10. Interestingly, after 6 h of treatment with IL-1α total APP levels were elevated, with sAPPα increased after 48 h (Bandyopadhyay et al., 2006). However, it is important to consider that ADAM10 activity is implicated in cancer progression and thus, this cell line may not be generalisable across more physiological models (Smith et al., 2020). Exposure of astrocytes to cytokines may also act in tandem with low concentrations of Aβ to promote a reactive state, possibly creating a distinct neurotoxic astrocyte profile (LaRocca et al., 2021). Treatment of astrocytes with IL-1β has been shown to induce extracellular vesicle release, which contain altered cargo proteins compared to resting astrocyte-derived vesicles. The application of IL-1β stimulated astrocyte vesicles onto neuronal cultures was able to significantly increase neuronal Aβ generation, however, direct treatment of neuronal cultures with IL-1β only, failed to show a similar increase (Li et al., 2020). This highlights the potential interplay between reactive states of astrocytes and APP processing. Interestingly, Aβ oligomer treatment, which has been shown to induce proinflammatory conditions, can alter ADAM10 localisation and this in turn, may indicate a different mechanism of action of Aβ on ADAM10 activity (Marcello et al., 2019). Treatment with Aβ has further been shown to disrupt cellular calcium flux, decreasing ADAM10 expression (Grolla et al., 2013). In fact, this may be a concentration dependent mechanism with low doses of Aβ acting synergistically with cytokines to induce astrocyte reactivity. Thus, high doses of Aβ used in many cell studies may alter cell functions non-physiologically (LaRocca et al., 2021). Inhibiting astrogliosis can accelerate Aβ accumulation in AD mice (Kraft et al., 2013) indicating a protective role for astrocyte reactivity. Further, reactive astrocytes can help to clear Aβ, release trophic factors, regulate autophagy, and maintain redox balance by upregulating antioxidant systems (Chun and Lee, 2018). However, the progression toward a chronic state of reactive astrocytes in aging and disease, perhaps related to duration of disease, can be detrimental and could contribute to a persistent neuroinflammation and the presence of atrophic astrocytes in AD (Preman et al., 2021). Excessive accumulation of Aβ can also induce astrocyte reactivity. This can lead to dysregulation of calcium dynamics, altered neuron–glia communication and impaired synaptic transmission (Preman et al., 2021). Neuronal dystrophy and lowered axonal growth which is associated with Aβ accumulation may in part be mediated by impaired astrocytic functions, as evidenced in co-cultures of Aβ-treated astrocytes and neurons (Monnerie et al., 2005). In addition, Aβ may interact with astrocytes to reduce phagocytosis and the clearance of dystrophic synapses leading to neuron functional deficits (Afridi et al., 2020; Sanchez-Mico et al., 2021). Thus, healthy functional astrocytes may promote non-amyloidogenic APP processing by ADAM10 and increase neuronal plasticity which could be neuroprotective in AD (Lopes et al., 2022).

### Lipid transport and ADAM10 in astrocytes

The synthesis and transport of lipids, in particular cholesterol, is necessary for maintaining neuronal function and is primarily supplied by glial cells in the form of Apolipoprotein E (APOE; Zhang and Liu, 2015). The brain holds up to 25% of the body’s cholesterol and levels are tightly regulated for normal physiological function (Feringa and Van der Kant, 2021). In AD, the accumulation of cholesterol may drive pathological processes. Evidence of lipid transport deficits in AD pathology are supported by the increased risk of AD associated with genes highly expressed by glial cells, such as APOE, Clusterin, Feritin, and ABCA7, all of which impact lipid transport (Preman et al., 2021; Picataggi et al., 2022). Of these genes, carrying one or two copies of the APOE Ɛ4 allele gives the greatest risk for developing late-onset AD. This is opposed to the most common APOE Ɛ3 alleles, or APOE Ɛ2, which is associated with a reduced risk of AD. Interestingly, astrocytes produce the majority of APOE in the central nervous system (Williams et al., 2020) and carrying APOE Ɛ4 significantly impacts astrocytic functions through multiple pathways, leading to impaired functioning. APOE Ɛ4 astrocytes display dysregulated lipid transport leading to lipid accumulation and impaired cholesterol efflux, when compared to APOE Ɛ3 astrocytes (de Leeuw et al., 2022; Lindner et al., 2022), and this is coupled with altered metabolic flux. Similarly, APOE Ɛ4 astrocytes have perturbed autophagy and mitophagy, and increased cytokine production in an allele dependent manner (APOE Ɛ4 > Ɛ3 > Ɛ2; de Leeuw et al., 2022; Eran and Ronit, 2022). This has been linked to increased amyloid pathology through astrocyte specific APOE reduction in mice (Mahan et al., 2022), although there is convening evidence that the link between APOE Ɛ4 and deficits in AD are initially amyloid independent. Astrocyte-derived cholesterol has been shown to be a key regulator of neuronal Aβ accumulation in neurons, by the transfer of APOE (Wang et al., 2021). This enrichment of neuronal membrane cholesterol leads to the increased generation of Aβ (Wang et al., 2021). The enzymes responsible for the liberation of Aβ from APP, BACE-1 and γ-secretase, are known to reside favorably in lipid rafts (Vetrivel and Thinakaran, 2010). Due to this, it is hypothesized that lipid clustering may increase Aβ production. This, linked with the observed accumulation of cholesterol in the brains of people with AD and the associated increased risk of carrying an Apolipoprotein-E (ApoE) ε4 allele, suggests cholesterol dyshomeostasis is a contributing factor toward AD (Feringa and Van der Kant, 2021). Cholesterol transport can also impact ADAM10 activity. Low cellular cholesterol induced by treatment with statins has been reported to elevate ADAM10 expression and promote sAPPα production (Kojro et al., 2001). Similarly, methyl-β-cyclodextrin treatment in COS-7 cells, stimulated IL-6 receptor shedding by ADAM10, indicating ADAM10 activity can be promoted under low cholesterol conditions (Matthews et al., 2003). Interestingly, it does not appear that elevated cholesterol has a negative effect on ADAM10 activity, in fact, inhibiting cholesterol transport can increase both α- and β-cleavage of APP in astrocytes and increase their secretion into astrocyte-derived exosomes (Yang et al., 2017; Wu et al., 2021).

## Discussion

There is significant interest in further understanding the role of astrocytes and glial cells in AD pathology. ADAM10 has been identified as a potential therapeutic target in the treatment of AD, with the hypothesis that increasing ADAM10 activity may slow disease progression. Increased ADAM10 activity is also associated with reactive gliosis which itself has been identified in the AD brain. Thus, ADAM10 activity may be differentially regulated between acute and chronic inflammatory states, whereby ADAM10 is upregulated acutely before a gradual decline in function over time. Equally, it is possible that ADAM10 activity is regulated by independent mechanisms between different cell types, possibly mediated by multiple TSPAN repertoires. While gliosis may increase astrocyte ADAM10 activity, it may have an inhibiting effect on neuronal ADAM10 activity. However, this is speculative and an area for future investigation. Of note, exercise, which is known to reduce the risk of AD, can reduce astrocyte reactivity and stimulate ADAM10 activity (Elsworthy et al., 2022). The mechanisms by which exercise might reduce the risk of AD is worthy of future investigation, both related to, and independent of the regulation of ADAM10 activity in the brain (Zhang et al., 2018, 2019). Overall, it is clear that more evidence is needed to understand the multifunctional role of astrocytes in the brain in order to aid our understanding of how they might contribute to AD pathology. The advancement in cell modeling of physiologically credible astrocytes may pave the way for more mechanistic research (Hill et al., 2016). Much of the available data on acute astrocyte reactivity points toward a potentially neuroprotective effect, linked to increase ADAM10 activity and elevated sAPPα release. However, in AD chronic neuroinflammation and glial reactivity points toward a much less favorable outcome.

## Author contributions

RE and CD were responsible for the first draft and figure design. EH and SA contributed to the editing and finalization of the review. All authors were involved in the initial conception and development of the review and contributed to the article and approved the submitted version.

## Funding

Open access publication fees were funded by the University of Birmingham.

## Conflict of interest

The authors declare that the research was conducted in the absence of any commercial or financial relationships that could be construed as a potential conflict of interest.

## Publisher’s note

All claims expressed in this article are solely those of the authors and do not necessarily represent those of their affiliated organizations, or those of the publisher, the editors and the reviewers. Any product that may be evaluated in this article, or claim that may be made by its manufacturer, is not guaranteed or endorsed by the publisher.
